# A 5-year Retrospective Study of Women with Postpartum Psychiatric Disorders Hospitalized in the Clinical Hospital of Psychiatry, Chisinau, Republic of Moldova

**DOI:** 10.1192/j.eurpsy.2025.2431

**Published:** 2025-08-26

**Authors:** L. Sanduleac, I. Deliv, V. Tapes, I. Cosciug

**Affiliations:** 1 Mental Health, Clinical Psychology and Psychotherapy Department, University of Medicine and Pharmacy “Nicolae Testemițanu”, Chisinau, Moldova, Republic of

## Abstract

**Introduction:**

The postpartum period is critical for the onset of mood disorders. Three primary postpartum affective disorders are recognized: baby blues, postpartum depression, and postpartum psychosis, each differing in prevalence, presentation, and treatment. Obstetric factors like primiparity, complications during pregnancy and birth, Cesarean sections, and preterm births are associated with increased neuropsychiatric risks during the puerperium.

**Objectives:**

This study analyzes the demographic, clinical, and obstetric factors linked to postpartum psychiatric disorders and case management in women hospitalized at the Hospital of Psychiatry in Chisinau, Moldova, over a five-year period.

**Methods:**

A longitudinal, retrospective study was conducted, reviewing medical records of 35 women hospitalized between 2019 and 2024. A literature review was also performed to identify relevant obstetric and clinical risk factors.

**Results:**

Patients ranged from 20 to 41 years old, with an average age of 29.8 years ±1,5 years. Out of reviewed cases, 42.9% were from urban areas, 25.7% had a family history of psychiatric illness, and 54.3% developed postpartum psychosis after their first birth, suggesting primiparity as a key risk factor. Psychosis onset ranged from three days to two months postpartum in 45.7% of cases, with 37.1% showing symptoms within the first two weeks. Comorbidities included cardiovascular diseases (20.0%), digestive disorders (14.3%), renal issues (8.6%), and autoimmune conditions (2.9%). Severe mental and behavioral disorders (F53.1) were diagnosed in 74.3% of cases, while 25.7% had milder forms (F53.0). First-time hospitalizations accounted for 88.6%, but 48.6% had prior hospitalizations, with later diagnoses evolving into paranoid schizophrenia, schizotypal disorder, and recurrent depression. Psychological consultation was provided in 48.6% of cases, using tools like PHQ-9, TAG, MMPI, BDI. Treatment varied significantly, with antipsychotics, antidepressants, and benzodiazepines prescribed, yet no standardized protocol was followed.

**Conclusions:**

Primiparity is a significant risk factor for postpartum psychiatric disorders, with early onset often occurring within the first two weeks. This underscores the critical need for prompt recognition and intervention. Comorbid disorders add complexity to patient management. The variability in pharmacological and nonpharmacological treatment highlights a gap in evaluation and case management, potentially delaying timely diagnosis and treatment and it emphasizes the need for consistent and standardized guidelines. The evolving nature of diagnoses, reinforces the importance of ongoing monitoring, psychoeducation, and psychological support throughout the postpartum period.

**Disclosure of Interest:**

None Declared

